# Mice lacking melatonin MT2 receptors exhibit attentional deficits, anxiety and enhanced social interaction

**DOI:** 10.1177/02698811211032439

**Published:** 2021-07-24

**Authors:** David M Thomson, Emma J Mitchell, Rebecca L Openshaw, Judith A Pratt, Brian J Morris

**Affiliations:** 1 Strathclyde Institute of Pharmacy and Biomedical Sciences, University of Strathclyde, Glasgow, UK; 2 Institute of Neuroscience and Psychology, College of Medical, Veterinary and Life Sciences, University of Glasgow, Glasgow, UK

**Keywords:** Melatonin receptor, attention, cognition, social interaction, 5-HT2C receptor, sex-dependent

## Abstract

**Background:**

Aside from regulating circadian rhythms, melatonin also affects cognitive processes, such as alertness, and modulates the brain circuitry underlying psychiatric diseases, such as depression, schizophrenia and bipolar disorder, via mechanisms that are not fully clear. In particular, while melatonin MT1 receptors are thought primarily to mediate the circadian effects of the hormone, the contribution of the MT2 receptor to melatonin actions remains enigmatic.

**Aims:**

To characterise the contribution of MT2 receptors to melatonin’s effects on cognition and anxiety/sociability.

**Methods:**

Mice with a genetic deletion of the MT2 receptor, encoded by the *Mtnr1b* gene, were compared with wild-type littermates for performance in a translational touchscreen version of the continuous performance task (CPT) to assess attentional processes and then monitored over 3 days in an ethological home-cage surveillance system.

**Results:**

*Mtnr1b* knockout (KO) mice were able to perform at relatively normal levels in the CPT. However, they showed consistent evidence of more liberal/risky responding strategies relative to control mice, with increases in hit rates and false alarm rates, which were maintained even when the cognitive demands of the task were increased. Assessment in the home-cage monitoring system revealed that female *Mtnr1b* KO mice have increased anxiety levels, whereas male *Mtnr1b* KO mice show increased sociability.

**Conclusions:**

The results confirm that the MT2 receptor plays a role in cognition and also modulates anxiety and social interactions. These data provide new insights into the functions of endogenous melatonin and will inform future drug development strategies focussed on the MT2 receptor.

## Introduction

Melatonin (MT) receptors are G protein-coupled receptors with highly restricted patterns of expression. MT1 receptors (encoded by the *MTNR1A* gene) are expressed in the vasculature, the pancreas and the pars tuberalis of the pituitary, and in the brain are enriched in the dentate gyrus and the suprachiasmatic nucleus of the hypothalamus, where MT1 receptors play a well-characterised role in the regulation of circadian rhythmicity (Ng et al., 2017). MT2 receptors (encoded by the *MTNR1B* gene) are also found in the pancreas. Indeed, in genome-wide association studies, human sequence variations in the in *MTNR1B* gene have been strongly and robustly associated with altered resting plasma glucose levels, and with maternal influences on foetal development (Horikoshi et al., 2013). There is growing interest in the possibility that MT receptors represent promising targets for the treatment of psychiatric disorders (Comai and Gobbi, 2014; Dubocovich, 2007; Kishi et al., 2019; Liu et al., 2016). For example, agomelatine is a novel, clinically effective antidepressant/anxiolytic combining antagonism of 5HT2C receptors with agonist activity at MT1/MT2 receptors (Taylor et al., 2014).

In the brain, MT2 receptors are located primarily in the CA3 region of the hippocampus and the thalamic reticular nucleus (TRN) (Lacoste et al., 2015; Ng et al., 2017; Ochoa-Sanchez et al., 2011). This distribution pattern suggests an important role in a wide range of cognitive functions. It is well established that the CA3 region of the hippocampus involved in learning and memory. The TRN has a key role in regulating thalamocortical function and has been proposed to act as an ‘attentional searchlight’ (Crick, 1984), focussing sensory information to facilitate salient stimuli and suppress irrelevant stimuli. This concept is supported by studies in primates in which TRN activity is modified by shifts of visual attention (McAlonan et al., 2006) and in mouse optogenetic studies in a cross-modal (visual and auditory) divided attention task (Wimmer et al., 2015). Importantly, these optogenetic studies showed an engagement of TRN subnetworks to enable optimal performance, whereby distinct patterns of TRN firing were dependent on the target sensory modality, consistent with a gating role of the TRN during selective attention. While the ‘sensory’ regions of the TRN have been explored in attentional tasks, there is a lack of knowledge of the role of ‘cognitive’-related TRN inputs from the prefrontal cortex (PFC) and mediodorsal thalamus in tests of cognitive flexibility and working memory (Pratt and Morris, 2015). The TRN is also involved in the generation of gamma oscillations (Macdonald et al., 1998; Pinault and Deschênes, 1992), which are linked to cognitive processes (Colgin et al., 2009; Osipova et al., 2006).

Evidence has accumulated that dysfunction of the TRN may play a crucial role in psychiatric disease aetiology (Ferrarelli and Tononi, 2011; Pratt and Morris, 2015). The localisation of MT2 receptors in the TRN may therefore be particularly relevant for understanding the role of MT in the CNS, and in particular, potential pro-cognitive effects (Wade et al., 2014). A non-selective MT1/MT2 agonist (ramelteon) was reported to improve cognitive function in a small cohort of schizophrenia patients (Shirayama et al., 2014). Conversely, ramelteon reportedly impaired cognitive function in a small group of normal volunteers (Miyata et al., 2015), similar to earlier reports with melatonin (Dollins et al., 1993), so there is a lack of clarity on the cognitive actions of combined MT1/MT2 agonists in humans.

In mice, an MT2 receptor agonist (UCM765) decreased anxiety in novel environments (elevated plus maze test, novelty suppressed feeding test and open-field test), increased non-rapid eye movement (NREM) sleep, and also increased TRN neuron firing (Comai and Gobbi, 2014; Comai et al., 2020; Ochoa-Sanchez et al., 2011, 2012), supporting the link between MT2 receptors and NREM sleep. Mice lacking both MT1 and MT2 receptors are hyperactive, with enhanced performance in a simple maze-based cognitive task (spontaneous alternations in a Y maze) (O’Neal-Moffitt et al., 2014). Mice lacking MT2 receptors alone reportedly show decreased NREM sleep, increased wakefulness (Comai et al., 2013) and reduced habituation in the elevated plus maze test of anxiety task (Larson et al., 2006). A role for MT2 receptors in reward processing has also been proposed (Clough et al., 2014; Hutchinson et al., 2012).

Despite great interest in understanding the central effects of melatonin, and drugs acting on melatonin receptors, the contribution of MT2 receptors to these actions remains unclear. In this study, we tested the hypothesis that mice lacking MT2 receptors would show deficits in cognitive tests of attentional capacity and cognitive flexibility (inhibitory control) and altered activity patterns in a low-stress environment by continuous monitoring of group housed animals in a home-cage setting. Importantly, we employ a translationally relevant mouse touchscreen task designed to be analogous to the common human continuous performance task (CPT) to measure attentional performance, including the ability to respond to ‘target’ visual pattern stimuli, to withhold responses to ‘non-target’ stimuli and to respond to task manipulations of varying cognitive load (Kim et al., 2015).

## Materials and methods

Mice with targeted deletion of the *Mtnr1b* gene (encoding the MT2 receptor), on C57Bl/6N background, were obtained under the knockout (KO) mouse project from MRC Harwell (Oxfordshire, UK). Note that the C57Bl/6N substrain does not have the *Aanat* mutation, present in the C57Bl/6J substrain, which compromises melatonin synthesis (e.g., see GenBank: BC119139.1; Transcript: MGP_C57BL6NJ_T0034145.1). Heterozygote breeding pairs were used to generate KO and wild-type (WT) control mice (*n* = 12/group), with 50% male and 50% female for each group. All animals were food restricted to within 85% of free feeding weight according to age and group housed in either groups of two or three animals per cage. The holding room was maintained under a reverse 12 h day/night cycle (lights off at 10 am). All studies were approved by the institution’s Animal Welfare and Ethical Review Body.

### Continuous performance task

Sessions occurred once daily, typically between 10.30 am and 2.30 pm, 5 days/week. *Mtnr1b* KO mice appeared overtly normal and exhibited similar growth curves to WT mice. On commencement of the training protocol for CPT studies, the average weights were 29.3 g for WT males, 29.8 g for KO males, 22.5 g for WT females and for 23.8 g KO females. Mouse touchscreen operant boxes (Campden Instruments Ltd. with ABET II touch software) (Loughborough, UK) had the main touchscreen masked apart from three horizontal apertures, allowing access to three discrete sections of the touchscreen. The task stimulus was presented in the centre panel, with the two flanking panels used for distractor stimuli during the distractor session. Reward (Yazoo™ strawberry milkshake – 70 µl) (FrieslandCampina, London, UK) was provided for correct responses, when a tone was also played and the food reward hopper illuminated. While ‘S+’ stimuli were rewarded, ‘S−’ responses triggered a correction phase, where the stimulus was repeatedly presented until a correct withholding of a response allowed an increment onto the next trial.

**Figure 1.**
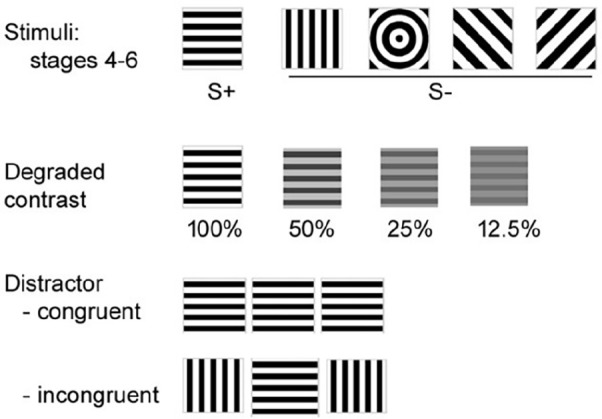
Stimuli used during the CPT.

Training stages were conducted as described by Kim et al. (2015). Stimuli used are shown in Figure 1. To probe cognitive function further, mice were tested for performance with a degraded stimulus, with flanking distractor stimuli (Figure 1), and with variable stimulus duration. For the last, the stimulus duration for S+ presentation was varied across three stimulus durations of 1 s, 0.6 s and 0.2 s with a probability of 33% each trial type. All other variables matched the stage 6 trial set.

The following main measures were recorded: ‘Hit’ – correct response with a touch to the S+ stimulus within the limited hold period; ‘Miss’ (omission) – failure to respond to a S+ stimulus; ‘Mistake’ (false alarm) – incorrect response to a S− stimulus and ‘Correct rejection’ – successful withholding of a response to the S− stimulus. From these basic measures, a series of compound measures were constructed as with the human CPT: hit rate (HR) – the rate of response to correct S+ stimulus – HR = Hit/(Hit+Miss); false alarm rate (FAR) – the rate of response to the S− stimulus – FAR = False alarm/(False alarm+Correct rejection); sensitivity index (SI) – the perceptual discriminability between the S+ and S−, that is, higher values indicate better visual discrimination (Kim et al., 2015) – SI = (HR−FAR)/(2(HR+FAR)−(HR+FAR)^2^) and responsivity index (RI) – the criterion or willingness to make responses, for example, conservative = low RI values or liberal = high RI values (Kim et al., 2015, see erratum for correct formula) – RI = (HR+FAR−1)/(1−(HR−FAR)^2^). This is effectively the converse of the LnBeta index used clinically, where low vales indicate less conservative, more liberal strategies.

### Home-cage monitoring

Subjects were 24 mice as above, aged 21–28 weeks during data acquisition. A radiofrequency identification (RFID) transponder was implanted subcutaneously under isoflurane anaesthesia, and a minimum of 24 h later, groups of three mice were transferred to a Home-Cage Analyser (Actual Analytics Ltd., Edinburgh, UK). After an initial 1.5 h prior to habituate, mice were monitored for 72 h (recording commenced at 10 am – onset of dark phase) (Bristow et al., 2020; Mitchell et al., 2020b). One cage (three WT females) was subsequently excluded from the analysis due to technical issues. Data output included, for 30 min time bins: total distance travelled (mm), total number of antenna transitions, separation (mean Euclidean distance to closest cage mate (mm)) and isolation (time spent >100 mm apart from cage mates (s)).

### Statistical analysis

There is a growing awareness that the addition of approaches that are complementary to null-hypothesis significance testing, such as Estimation statistics and Bayesian analysis, is often beneficial for the interpretation of the biological significance of reported effects. Hence, we have included these strategies in this piece of work. In addition to ANOVA analysis, for the main hypothesised effects, figures also show effect size estimation (estimationstats.com), with mean differences shown as Gardner–Altman estimation plots (Calin-Jageman and Cumming, 2019; Ho et al., 2019). For one sample and unpaired *t*-tests, we also show Bayes factors, estimated using JASP (Quintana and Williams, 2018) (The JASP team, Amsterdam, Netherlands, https://jasp-stats.org), with default (Cauchy) priors. The Bayes factors were robust against choice of priors (JASP) in each case. Bayes factor magnitudes were interpreted by standard practice in life sciences research (Lee and Wagenmakers, 2013).

## Results

### Continuous performance task

**Figure 2.**
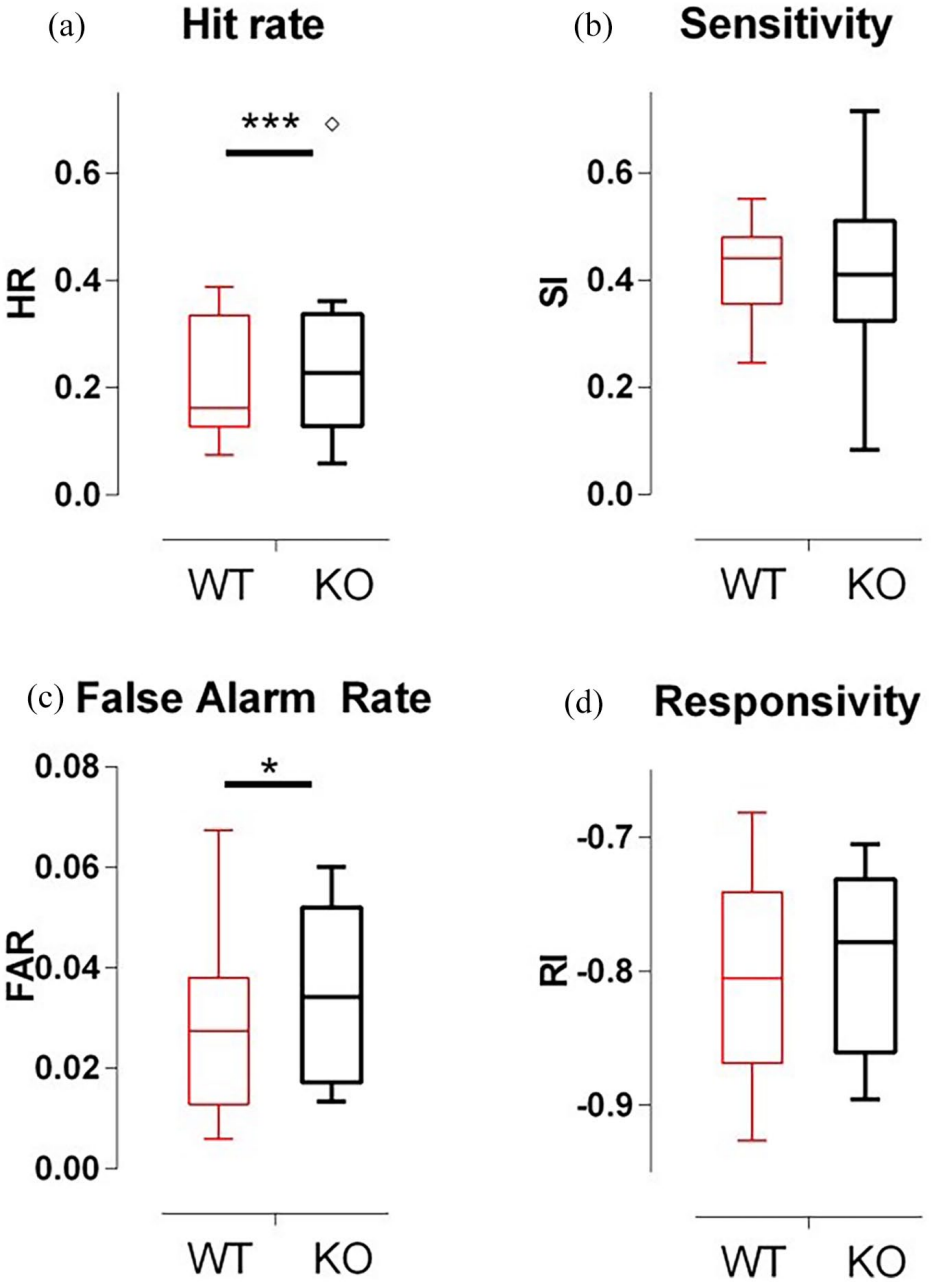
CPT proficiency of WT and Mtnr1b KO mice after full acquisition of task (stage 6): (a) hit rate, (b) sensitivity, (c) false alarm rate and (d) responsivity. Data were collected over five consecutive days. ANOVA (genotype and sex): effect of genotype *F*(1,114) = 13.05, *p* = 0.001 (a), *F*(1,114) = 0.92, ns (b), *F*(1,114) = 4.08, *p* = 0.047 (c) and *F*(1,114) = 2.90, *p* = 0.092 (d). Bars show median and interquartile range (sexes pooled for visualisation), with ‘Tukey’ whiskers and open symbols for outliers. ANOVA: analysis of variance; CPT: continuous performance task; KO: knockout; WT: wild type. *p < 0.05. ***p < 0.001 (ANOVA main effect).

*Mtnr1b* KO mice acquired the CPT at an equivalent rate to WT control mice, with no significant differences between the groups as they passed through stages 1–6 of task acquisition. Once at stage 6, *Mtnr1b* KO mice showed largely normal levels of performance over 5 days of baseline testing, except that the frequency of correct responses and false alarm responses was elevated (Figure 2(a) and (c)). By comparison, measures of sensitivity and reaction time were broadly equivalent to controls (Figure 2(b) and (d)). The total number of trials completed was also not significantly different in *Mtnr1b* KO mice (data not shown).

**Figure 3.**
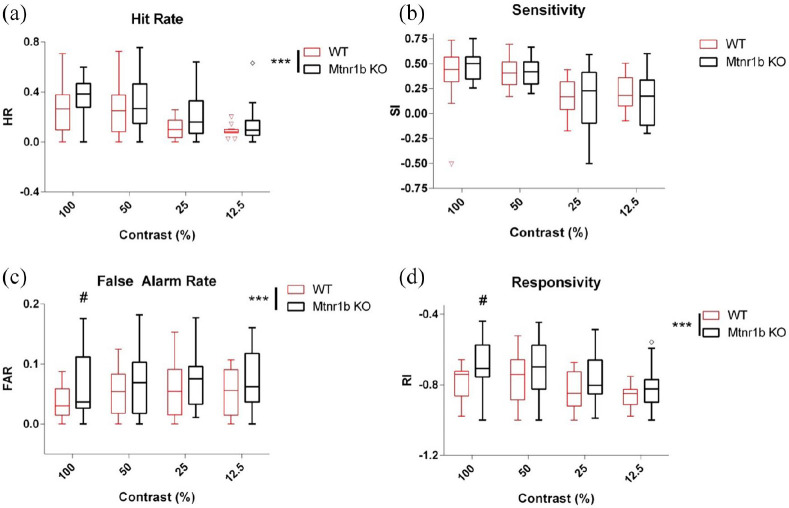
CPT proficiency of WT and Mtnr1b KO mice with decreased stimulus contrast: (a) hit rate, (b) sensitivity, (c) false alarm rate and (d) responsivity. ANOVA: effect of contrast *F*(3,95) = 28.50, *p* < 0.001 (a), *F*(3,90) = 14.45, *p* < 0.001 (b), *F*(3,95) = 1.77, ns (c), *F*(3,95) = 17.85, *p* < 0.001 (d), effect of genotype *F*(1,95) = 18.82, *p* < 0.001 (a), *F*(1,90) = 0.27, ns (b), *F*(1,95) = 15.43, *p* < 0.001 (c) and *F*(1,95) = 32.46, *p* < 0.001 (d). Sex was also included as an additional factor in the analysis. Bars show median and interquartile range, with ‘Tukey’ whiskers and open symbols for outliers. ANOVA: analysis of variance; CPT: continuous performance task; KO: knockout; WT: wild type. ***p < 0.001 (ANOVA main effect). #*p* < 0.05 versus corresponding WT group, post hoc Tukey’s test.

**Figure 4.**
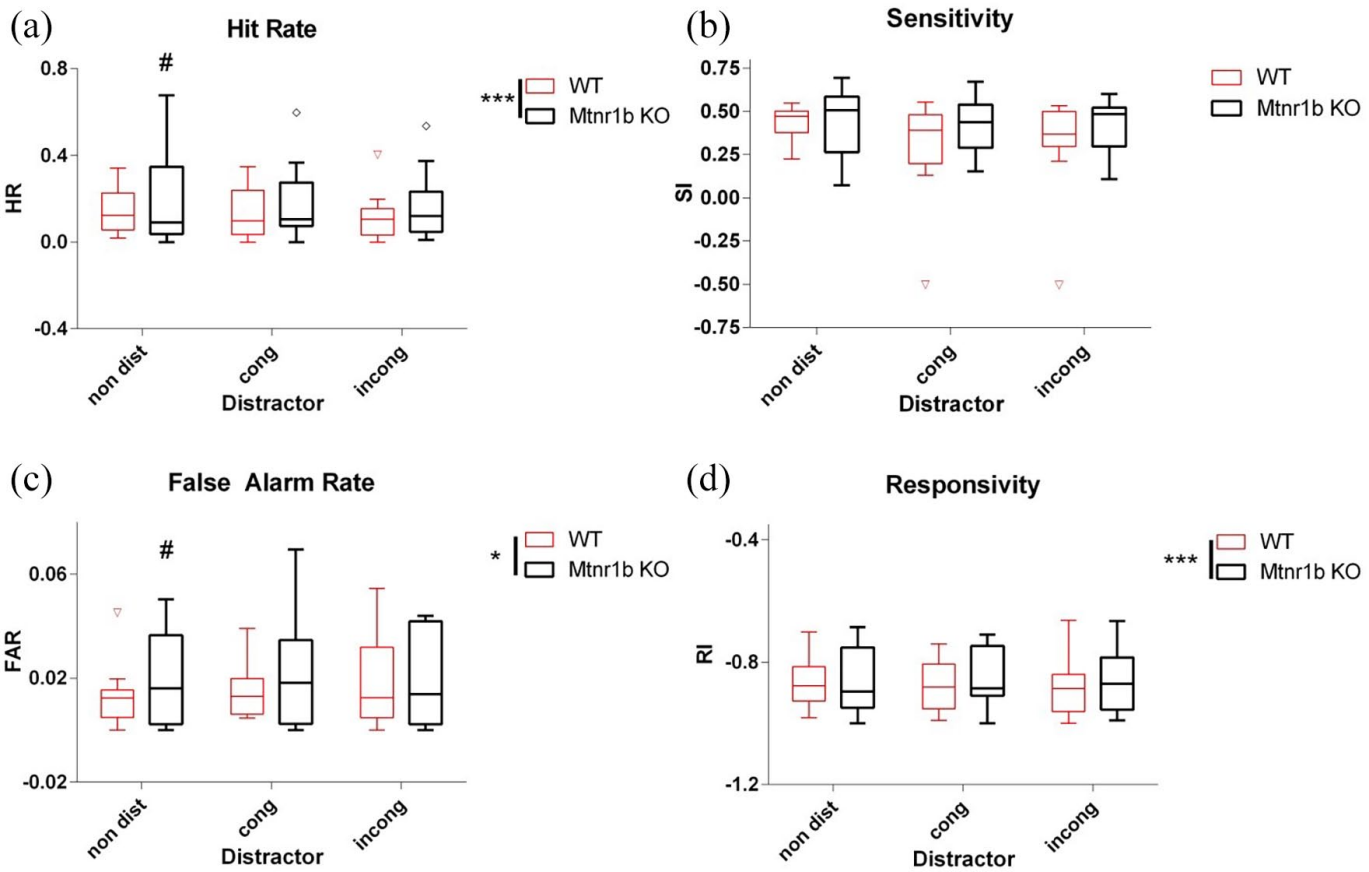
CPT proficiency of WT and Mtnr1b KO mice without (non-dist) or with a distractor stimulus (congruent (cong) or incongruent (incong)): (a) hit rate, (b) sensitivity, (c) false alarm rate and (d) responsivity. ANOVA: effect of distractor *F*(2,71) = 3.26, *p* = 0.048 (a), *F*(2,68) = 2.08, ns (b), *F*(2,71) = 0.28, ns (c), *F*(2,71) = 1.02, ns (d), effect of genotype *F*(1,71) = 23.34, *p* < 0.001 (a), *F*(1,68) = 3.77, *p* = 0.059 (b), *F*(1,71) = 5.06, *p* = 0.030 (c), *F*(1,71) = 12.62, *p* < 0.001 (d). Sex was also included as an additional factor in the analysis. Bars show median and interquartile range, with ‘Tukey’ whiskers and open symbols for outliers. ANOVA: analysis of variance; CPT: continuous performance task; KO: knockout; WT: wild type. *p < 0.05. ***p < 0.001 (ANOVA main effect). #*p* < 0.05 post hoc Tukey’s test.

The parameters of the task were then manipulated to increase cognitive load. Reducing the contrast of the stimulus led to reduced HRs, perceptual sensitivity and responsivity without affecting FARs (Figure 3). The introduction of a distractor slightly reduced HRs without significantly affecting the other parameters (Figure 4). In both cases, the increased FARs and impulsivity of the *Mtnr1b* KO mice were maintained despite the increased difficulty of the task (no significant genotype x contrast/distractor interaction) (Figures 3 and 4). For manipulations to increase cognitive load, sex effects were not detected in general, with the exception of FARs with variable stimulus duration (males > females, *F*(1,71) = 8.1; *p* = 0.007), and of responsivity with variable stimulus duration, altered contrast and inclusion of distractor (males > females, *F*(1,71) = 66.9, 17.2 and 130.3; *p* < 0.001, *p* < 0.001 and *p* < 0.001, respectively).

**Figure 5.**
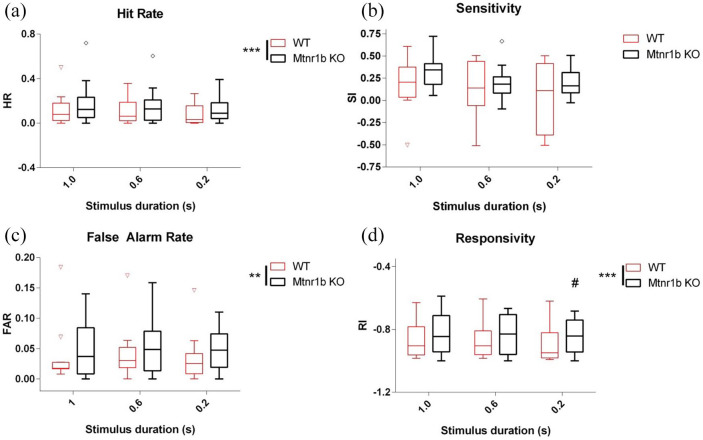
CPT proficiency of WT and Mtnr1b KO mice with variable stimulus duration: (a) hit rate, (b) sensitivity, (c) false alarm rate and (d) responsivity. ANOVA: effect of stimulus duration *F*(2,71) = 4.84, *p* = 0.013 (a), *F*(2,67) = 1.53, ns (b), *F*(2,71) = 1.57, ns (c), *F*(2,71) = 2.88, *p* = 0.067 (d), effect of genotype *F*(1,71) = 15.97, *p* < 0.001 (a), *F*(1,67) = 3.47, *p* = 0.07 (b), *F*(1,71) = 10.42, *p* = 0.002 (c), *F*(1,71) = 26.40, *p* < 0.001 (d). Sex was also included as an additional factor in the analysis. Bars show median and interquartile range, with ‘Tukey’ whiskers and open symbols for outliers. ANOVA: analysis of variance; CPT: continuous performance task; KO: knockout; WT: wild type. **p < 0.01. ***p < 0.001 (ANOVA main effect). #*p* < 0.05, post hoc Tukey’s test.

Decreasing the duration of the stimulus produced the anticipated decrement in HR in both genotype groups (Figure 5) and was similar to the effect of a distractor; in that, the other parameters were not significantly affected.

**Figure 6.**
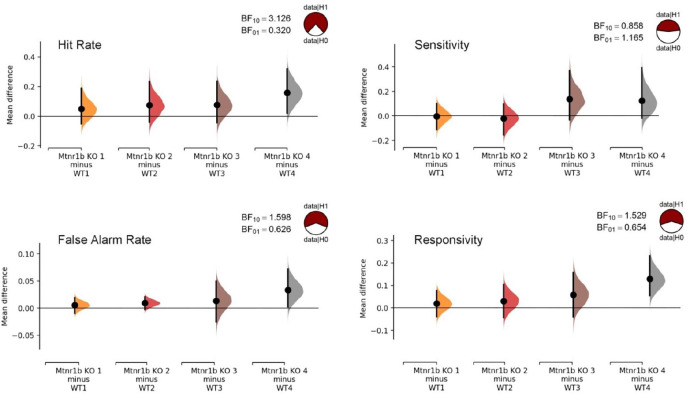
Robustness of attentional abnormalities in Mtnr1b knockout (KO). The mean differences are shown in the Cumming estimation plots for stage 6 (pair 1), and the standard parameter parts of the distractor (pair 2), variable stimulus duration (pair 3) and degraded stimulus (pair 4) stages. Each mean difference is plotted as a bootstrap sampling distribution. Mean differences are depicted as dots; 95% confidence intervals are indicated by the ends of the vertical error bars. Results of the Bayesian one-sample *t*-tests for the mean differences versus zero (Bayes factor computed with the default prior) are also indicated, along with ‘pizza’ plots depicting the odds of the data under the null versus alternative hypothesis. Apart from sensitivity, analysis confirms anecdotal to moderate confidence in the genotype effects.

The experiments with increased cognitive load, be it the presence of a distractor, or reduced stimulus duration or stimulus contrast, all incorporate a component of the session using the standard test conditions. To confirm the robustness of the cognitive abnormalities across the CPT test sessions, we directly compared the effect size of the genotype effects in stage 6 (before the increased cognitive load sessions) and in the standard condition components of the subsequent probe sessions (Figure 6). While no evidence for abnormal sensitivity was obtained, the effects on HR, FAR and responsivity were found to be consistent.

Overall, the CPT data indicate subtle attentional impairment in *Mtnr1b* KO mice, with a tendency to be more impulsive and less cautious in response selection.

### 24-h monitoring

**Figure 7.**
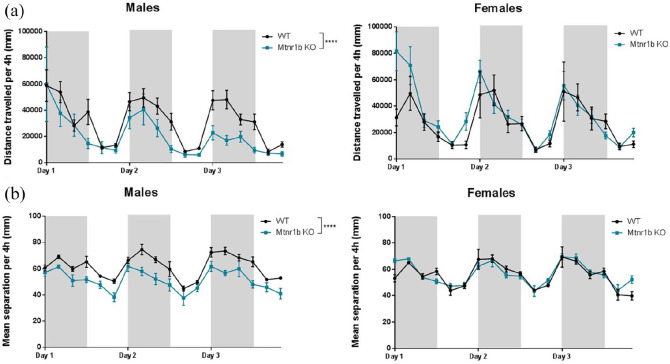
Circadian patterns of activity in home cage monitoring for males and females: (a) locomotor activity (distance travelled) shown in 4 h time bins. Main effects of sex (*F* (1, 459) = 8.46; *p* = 0.04), dark/light phase (*F* (1, 459) = 129.30; *p* < 0.0001) and 4 h time bin (*F* (16, 459) = 13.46; *p* < 0.0001) and two-factor interaction between genotype and sex (*F* (1, 459) = 33.76; *p* < 0.0001). The Tukey’s post hoc test revealed significant hypoactivity in male Mtnr1b knockout (KO) versus male wild-type (WT) (*p* < 0.0001). (no other interactions) and (b) social interaction (separation from cage mates) shown in 4 h time bins. Main effects of genotype (*F* (1, 459) = 26.46; *p* < 0.0001), dark/light phase (*F* (1, 459) = 43.43; *p* < 0.0001) and 4 h time bin (*F* (16, 459) = 90.03; *p* < 0.0001) and two-factor interaction between genotype and sex (*F* (1, 459) = 66.40; *p* < 0.0001). The Tukey’s post hoc test revealed significant reduced separation in male Mtnr1b KO versus male WT (*p* < 0.0001) (no other interactions).

The anticipated circadian variation in patterns of activity was observed, with all mice being substantially more active in the dark phase, as compared to the light phase (Figure 7).

There were differences in the pattern of activity between male and female *Mtnr1b* KO mice. Male *Mtnr1b* KO mice showed reduced locomotor activity compared to male WT mice, and this was increasingly apparent over time. They also exhibited less separation distance from their cage mates than WT controls. In contrast, distance travelled and separation distance were unchanged in female *Mtnr1b* KO mice compared to female WT controls.

**Figure 8.**
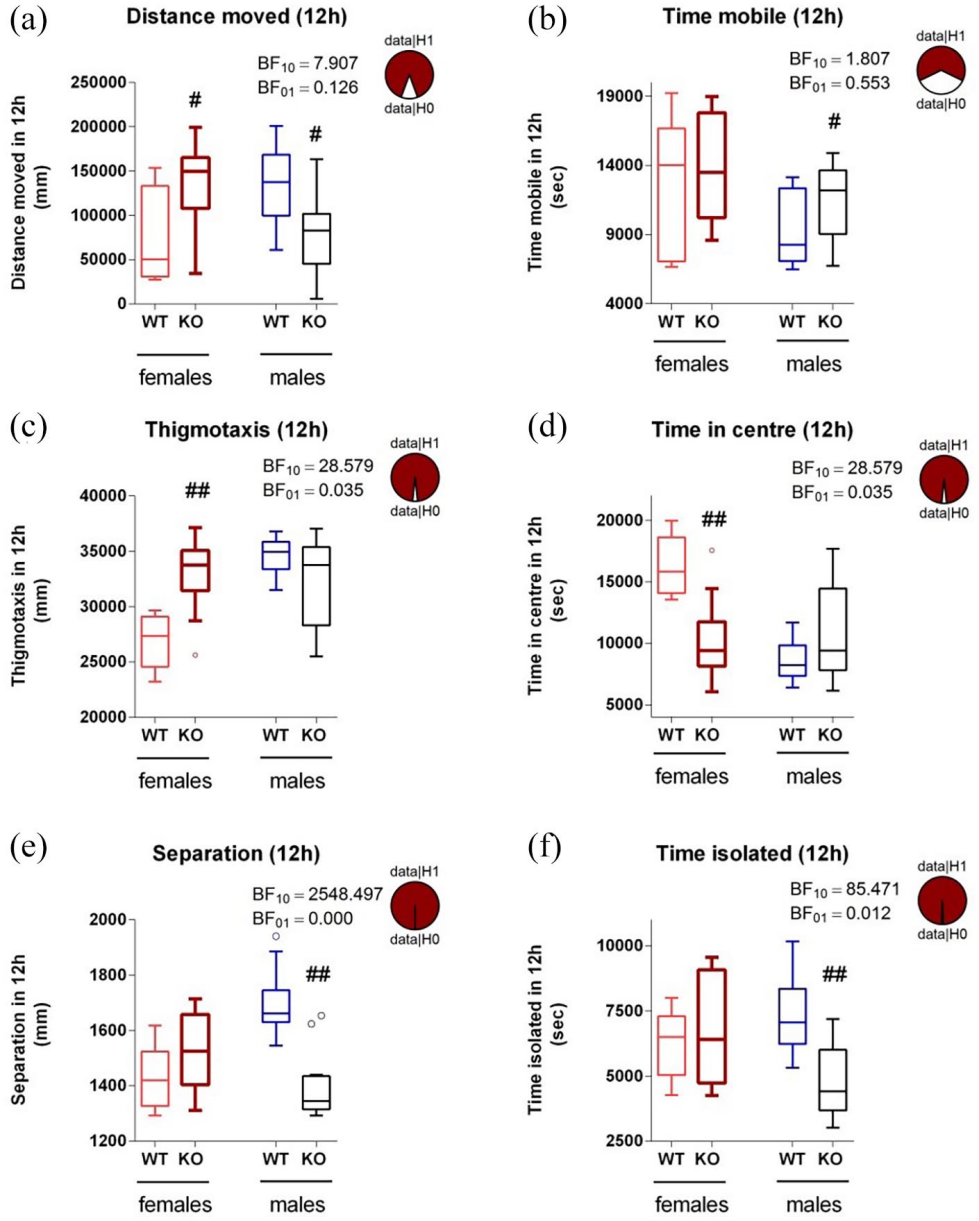
Locomotor, social and anxiety-related activity in the dark phase on days 2 and 3, assessed via home-cage monitoring, shown separately for males and females: (a) locomotor activity (distance travelled), (b) time spent mobile, (c) thigmotaxis, (d) time in centre zone, (e) social interaction (separation from cage mates) and (f) social interaction (time spent isolated from cage mates). Effect of genotype: *F*(1,125) = 0.11, ns (a), *F*(1,125) = 8.34, *p* = 0.005 (b), *F*(1,125) = 5.35, *p* = 0.023 (c), *F*(1,125) = 7.31, *p* = 0.008 (d), *F*(1,125) = 11.25, *p* = 0.001 (e), *F*(1,125) = 6.85, *p* = 0.010 (f), effect of sex *F*(1,125) = 0.09, ns (a), *F*(1,125) = 24.32, *p* < 0.001 (b), *F*(1,125) = 18.18, *p* < 0.001 (c), *F*(1,125) = 22.36, *p* < 0.001 (d), *F*(1,125) = 5.25, *p* = 0.024 (e) and *F*(1,125) = 1.08, ns (f). #*p* < 0.05, ##*p* < 0.01, post hoc Tukey’s test. Results of the Bayesian *t*-tests (Bayes factors were computed with the default prior) are also indicated, along with ‘pizza’ plots depicting the odds of the data under the null versus alternative hypothesis, for males only (a), (b), (e) and (f), or for females only (c) and (d). Analysis confirms moderate or strong to extreme confidence in the genotype effects.

When a deeper analysis of behaviour during the dark (most active) period was performed, it was confirmed that the *Mtnr1b* KO males were less active (in terms of distance moved) than WT controls, although time spent mobile was slightly increased (Figure 8). *Mtnr1b* KO male mice also appeared more sociable, spending more time in social interaction (shorter distance of separation and shorter time isolated from cage mates) than WT control males. These changes in social behaviour did not appear to result from altered anxiety levels as thigmotaxis and time spent in the centre of the cage was unchanged. Conversely, the female *Mtnr1b* KO showed clear evidence of an anxious phenotype, with increased thigmotaxis and reduced time spent in the centre of the cage but with no change in social behaviours (Figure 8).

## Discussion

*Mtnr1b* KO mice were overtly normal, but showed attentional deficits, and when group housed in a low-stress environment, there was increased sociability in males and increased anxiety in female mice. The results provide insight into the effects mediated by endogenous melatonin, out with its well-studied modulation of circadian rhythm.

There has been some debate about studying melatonin effects in C57Bl6 mice as compared to C3H/He mice. Indeed, mice of the C57Bl6J strain produce low levels of endogenous melatonin in the pineal and have lower plasma melatonin compared to the C3H/He inbred strain. Nevertheless, levels are similar in C57Bl6 mice to those in outbred strains (Gomez-Corvera et al., 2009; Goto et al., 1989; Vivien-Roels et al., 1998). The low pineal melatonin concentrations in C57Bl6J mice are proposed to be due to a synthetic enzyme mutation (Roseboom et al., 1998), although there is evidence that this mutation is not present in all tissues, and that other cells may contribute to measurable plasma melatonin levels (Conti et al., 2000; Gomez-Corvera et al., 2009) (but see also Kennaway, 2019). It is important that in this study the *Mtnr1b* KO mice used (from International Mouse Phenotyping Consortium), were produced on the C57Bl6N substrain (not the C57Bl6J strain), where this mutation does not occur, and C57Bl6N mice are known to produce melatonin from peripheral synthesis sites (Conti et al., 2000). Indeed, C57Bl6N, C57Bl6J and C3H-He strains show similar age-related patterns of circadian activity (Banks et al., 2015). It should also be remembered that genetic deletion of MT2 receptors is predicted to have effects even in the absence of any endogenous melatonin signalling. For example, the function of serotonin acting at MT2/5-HT2C receptor heteromers (Kamal et al., 2015) will be perturbed in Mtnr1b KO mice, and of course 5-HT2C receptors have a well-characterised role in anxiety and sociability (Heisler et al., 2007; Millan, 2005), and also in restraining impulsivity (Fletcher et al., 2013; Robinson et al., 2008). Hence, the disrupted behaviours observed in the *Mtnr1b* KO mice may in part relate to alterations in the functionality of MT2/5-HT2C receptor heteromers.

### Cognitive dysfunction in *Mtnr1b* KO mice

In the CPT, subtle alterations in performance were caused by the lack of MT2 receptors. The potential confound of metabolic factors arising from a role of MT2 receptors in glucose regulation is unlikely as cognitive testing is conducted when both genotypes are at equivalent weights on their respective growth curves. While in most cases, sensitivity of responding was unaffected, the *Mtnr1b* KO mice had a higher HR, but showed a robust propensity to make errors of commission (false alarms) and adopt a more liberal response strategy, compared to WT controls. The *Mtnr1b* KO mice were able to cope with the increased cognitive load as efficiently as WT mice. Their liberal responding strategies remained, however, whatever the difficulty of the task. Therefore, *Mtnr1b* receptors have a role in the inhibitory control mechanisms.

The manipulations introduced to increase cognitive load (reduced stimulus contrast, variable stimulus duration and introduction of a distractor) all produced the anticipated impairments in the CPT performance. Interestingly, reductions in HR appeared to be the most sensitive index of the increased difficulty (reductions in accuracy or sensitivity or both). These findings are similar to previous reports in mice (Hvoslef-Eide et al., 2018; Kim et al., 2015). The data are also similar to those observed clinically, emphasising the translational value of this paradigm. For example, decreased contrast (degraded stimulus) produces decreased sensitivity and decreased response bias in healthy control subjects (Chen et al., 1998; Hsieh et al., 2005). There was no evidence to suggest that the cognitive changes in *Mtnr1b* KO mice are related to altered sleep – circadian patterns of behaviour appeared grossly normal, and previous evidence has generally found no change in response bias after sleep loss (Horne et al., 1983).

It is of interest that anterior cingulate cortex lesions produce a remarkably similar impairment in mice (increased FARs and impulsivity, maintained with increasing cognitive load) (Hvoslef-Eide et al., 2018). The TRN is thought to play a key role in attentional processes, including a role in gating and gain control during selective attention (Guillery et al., 1998; Wimmer et al., 2015), and the projection from the cingulate to the TRN is proposed to contribute to this (Fitzgibbon and Kikuchi, 2011). Hence, the lack of MT2 receptors in the TRN may have contributed to the cognitive changes observed. The phenotype may also be relevant to impairments observed with psychiatric disease. Patients with schizophrenia tend to show reduced perceptual sensitivity relative to healthy controls, while response bias can be unaffected, increased or decreased (Hsieh et al., 2005; Ito et al., 1997; Liu et al., 1997; Mass et al., 2000; Seidman et al., 1998). However, patients with major depressive disorder show reduced response inhibition (Katz et al., 2010), and increased FARs have been detected (Cornblatt et al., 1989). Patients with bipolar disorder also show increased FARs and a more liberal response pattern in the CPT (Sax et al., 1995). A depression/bipolar disorder-related cognitive phenotype in mice lacking MT2 receptors is therefore of great interest with respect to ideas that melatonin agonists may be useful for treating depression or bipolar disorder (Alston et al., 2019; Kishi et al., 2019).

### Anxiety and locomotor activity in *Mtnr1b* KO mice

We tested to see whether ethological behaviours are disrupted in *Mtnr1b* KO mice. We choose to explore these behaviours in group-housed mice in a home-cage environment as (1) ethologically relevant behaviours can be measured simultaneously in the same mice, (2) the temporal aspects of behaviour can be monitored over several days and (3) mice are subject to less stress than in tests, such as the EPM and three-chamber test, where single animals are taken out of their home cage and placed in a novel environment (Bains et al., 2016; Mitchell et al., 2020a)

In relation to locomotor activity, the anticipated circadian variation in patterns of activity was observed, with male and female mice of both genotypes showing more activity in the dark phase as compared to the light phase. Of interest is the marked increase in locomotor activity immediately prior to the onset of the dark phase, which then subsides during the course of the dark period. We and others have noted this pattern of activity (Bains et al., 2016; Bristow et al., 2020), which may relate to anticipation of feeding and other dark phase activities.

Notably, male *Mtnr1b* KO mice, however, showed reduced locomotor activity compared to male WT mice, which was increasingly apparent over time. A deeper analysis of behaviour during the dark phase confirmed that distance moved was reduced in male *Mtnr1b* KO mice relative to WT mice, although the time spent mobile was increased. This is a very unusual combination of findings, suggesting that the male *Mtnr1b* KO mice are moving, but more slowly than WT mice. Speculatively, this may relate to engagement in other behaviours during this phase. However, these did not appear to result from altered anxiety levels as thigmotaxis and time spent in the centre of the cage was unchanged.

In contrast, we observed increased anxiety-related responses (decreased time in centre and increased thigmotaxis) in female *Mtnr1b* KO mice. Genetic deletion of the *Mtnr1b* gene in mice on a C3H/HeJ background also reportedly causes increased anxiety (Liu et al., 2017), despite the fact that mice (sex not specified) were single-housed, which can influence anxiety in mice (Voikar et al., 2005). It would be of interest to examine whether the exposure of *Mtnr1b* KO mice to other more stressful paradigms (such as the elevated plus-maze) would reveal sex-dependent effects on anxiety. A recent study (Comai et al., 2020) also reported an anxiety-like phenotype in male *Mtnr1b* KO mice (C3H/HeN background strain) that was limited to the light phase in one task and limited to the dark phase in another task. Hence, it is clear that MT2 receptors are involved in reducing anxiety, albeit with some complexity in how the anxiogenic phenotype becomes apparent.

A role for MT2 receptors in anxiety regulation is not unexpected. Agomelatine suppresses anxiety in rats (Papp et al., 2006; Regenass et al., 2018), as does melatonin (Bustamante-García et al., 2014; Comai and Gobbi, 2014; Papp et al., 2006). A novel selective MT2 agonist also has anxiolytic properties (Ochoa-Sanchez et al., 2012), so our data reinforce the concept that MT2 receptors modulate anxiety responses, and that activation of MT2 receptors may have therapeutic potential in anxiety disorders.

### Social dysfunction in *Mtnr1b* KO mice

The combination of reduced distance of separation and reduced isolation time, as observed in group housed male *Mtnr1b* KO mice over a sustained period in a low-stress environment, is strongly indicative of increased sociability. Genetic deletion of the *Mtnr1b* gene in mice on a C3H/HeJ background reportedly does not affect social interaction (in a standard three-chamber test) (Liu et al., 2017). The discrepancy with our data in terms of the social interaction may reflect differences in output measures of the three-chamber test, or, as noted above, the fact that testing was conducted in the light phase and in single-housed mice, possibly the sex of the mice (not specified), along with the possible additional contribution of blindness.

There has been particular interest in male-specific effects on social responsiveness, considering the increased incidence of autism spectrum disorders (ASD) in males compared to females. Our data, implicating melatonin and MT2 receptors in modulation of social behaviours in males are therefore of significant interest.

### Sex-dependent effects in *Mtnr1b* KO mice

The sex-dependent effects observed in the present study are intriguing. Previous studies of genetic deletion of the MT1 receptor have shown sex-dependent changes in terms of exploratory and anxiety measures in a novel environment in WT mice that were no longer apparent in MT1 receptor KO mice (Weil et al., 2006). Sex differences in spontaneous exploratory activity were apparent in singly housed MT1 KO mice, where behaviour was recorded over several days (Adamah-Biassi et al., 2014). These data are consistent with our data, in suggesting sex differences in the effects of melatonin signalling in the CNS. It is clear that in humans, there are differences between the sexes in terms of circadian melatonin fluctuations (Cain et al., 2010; Gunn et al., 2016), with women showing higher peak plasma levels. Assuming that this is also the case in the CNS, this implies that the behavioural effects of disrupting melatonin signalling in humans are likely to differ between the sexes. This provides some interesting context for our results showing that female *Mtnr1b* KO mice have increased anxiety levels, whereas male *Mtnr1b* KO mice show increased sociability.

The possibility of an interaction between sex hormones and melatonin in mediating sex differences in behaviour is a mute point. There is strong evidence that ovarian hormones can affect emotional and cognitive behaviours (ter Horst et al., 2012). In particular, stressors often affect females differently from males, which may be related to the stage of the oestrus cycle. In novel environments, such as the elevated plus-maze and the open field, female rodents generally exhibit less anxiety-like behaviours than males (ter Horst et al., 2012). In the context of the present study, where group-housed animals are monitored for 72 h in a low-stress home-cage environment, it is interesting to note that female *Mtnr1b* KO mice exhibited increased anxiety compared to males. However, it is worth noting that not all behaviours recorded in the present study showed sex-dependent effects. Taken together, our findings of increased anxiety but not social-related behaviours in female *Mtnr1b* KO mice support an interaction between melatonin and oestrogen in influencing some but not all behaviours. It is possible that melatonin plays both a neuroendocrine role (hypothalamic–pituitary–gonadal system) along with a neurotransmitter role to modulate particular behaviours. The precise neurobiological mechanisms that underpin this relationship to mediate sex-dependent changes in behaviours warrant further investigation.

### Neurobiological implications

We have been interested in the role of the TRN in the aetiology of psychiatric disease, and in the processes underlying cognitive activity in thalamocortical networks (Pratt and Morris, 2015). The TRN projects most extensively to the mediodorsal nucleus of the thalamus (MD), which itself projects massively to PFC and orbitofrontal cortex (OFC). Due to the high expression levels of MT2 receptors in the TRN, we consider it possible that many of the effects of *Mtnr1b* gene deletion that we report here reflect altered activity in this TRN–MD–PFC/OFC network. Consistent with this idea, direct inhibition of MD in rats decreases social behaviour and also decreases anxiety (Ferguson and Gao, 2018). Similarly, a minor suppression of MD activation of PFC or OFC results in cognitive impairment (Parnaudeau et al., 2013, 2015). It is also relevant that ketamine, attracting attention as a rapidly acting antidepressant, may act primarily on the TRN (Dawson et al., 2011; Pratt et al., 2017). Indeed, the cognitive impairment also observed with ketamine administration (Davis et al., 2021) is also likely to involve an action on the TRN. It would be of interest to directly manipulate MT2 receptors in components of the TRN–MD–PFC/OFC network in order to determine their involvement in the aforementioned behaviours.

Overall, the combination of impaired inhibitory control, anxiety and altered sociability is reminiscent of compromised MD–PFC connectivity (Ferguson and Gao, 2018). The data are consistent with the concept that TRN–MD–PFC/OFC network dysfunction can be caused by *Mtnr1b* gene deletion. Our observations add to an emerging view (Alston et al., 2019; Comai and Gobbi, 2014; Liu et al., 2016) that MT2 receptors offer a promising therapeutic target for mood disorders.

## References

[bibr1-02698811211032439] Adamah-Biassi EB Hudson RL Dubocovich ML (2014) Genetic deletion of MT1 melatonin receptors alters spontaneous behavioral rhythms in male and female C57BL/6 mice. Horm Behav 66: 619–627. 25200199 10.1016/j.yhbeh.2014.08.012 PMC4698802

[bibr2-02698811211032439] Alston M Cain SW Rajaratnam SMW (2019) Advances of melatonin-based therapies in the treatment of disturbed sleep and mood. Handb Exp Pharmacol 253: 305–319. 31123831 10.1007/164_2018_139

[bibr3-02698811211032439] Bains RS Cater HL Sillito RR , et al. (2016) Analysis of individual mouse activity in group housed animals of different inbred strains using a novel automated home cage analysis system. Front Behav Neurosci 10: 106. 27375446 10.3389/fnbeh.2016.00106 PMC4901040

[bibr4-02698811211032439] Banks G Heise I Starbuck B , et al. (2015) Genetic background influences age-related decline in visual and nonvisual retinal responses, circadian rhythms, and sleep. Neurobiol Aging 36: 380–393. 25179226 10.1016/j.neurobiolaging.2014.07.040 PMC4270439

[bibr5-02698811211032439] Bristow GC Thomson DM Openshaw RL , et al. (2020) 16p11 Duplication disrupts hippocampal-orbitofrontal-amygdala connectivity, revealing a neural circuit endophenotype for schizophrenia. Cell Rep 31: 107536. 32320645 10.1016/j.celrep.2020.107536

[bibr6-02698811211032439] Bustamante-García R Lira-Rocha AS Espejo-González O , et al. (2014) Anxiolytic-like effects of a new 1-N substituted analog of melatonin in pinealectomized rats. Prog Neuropsychopharmacol Biol Psychiatry 51: 133–139. 24495777 10.1016/j.pnpbp.2014.01.015

[bibr7-02698811211032439] Cain SW Dennison CF Zeitzer JM , et al. (2010) Sex differences in phase angle of entrainment and melatonin amplitude in humans. J Biol Rhythms 25: 288–296. 20679498 10.1177/0748730410374943 PMC3792014

[bibr8-02698811211032439] Calin-Jageman RJ Cumming G (2019) Estimation for better inference in neuroscience. eneuro 6: ENEURO.0205. 10.1523/ENEURO.0205-19.2019 PMC6709209 31453316

[bibr9-02698811211032439] Chen WJ Hsiao CK Hsiao LL , et al. (1998) Performance of the continuous performance test among community samples. Schizophr Bull 24: 163–174. 9502554 10.1093/oxfordjournals.schbul.a033308

[bibr10-02698811211032439] Clough SJ Hutchinson AJ Hudson RL , et al. (2014) Genetic deletion of the MT1 or MT2 melatonin receptors abrogates methamphetamine-induced reward in C3H/HeN mice. Physiol Behav 132: 79–86. 24813704 10.1016/j.physbeh.2014.04.049 PMC4698795

[bibr11-02698811211032439] Colgin LL Denninger T Fyhn M , et al. (2009) Frequency of gamma oscillations routes flow of information in the hippocampus. Nature 462: 353–357. 19924214 10.1038/nature08573

[bibr12-02698811211032439] Comai S De Gregorio D Posa L , et al. (2020) Dysfunction of serotonergic activity and emotional responses across the light-dark cycle in mice lacking MT(2) receptors. J Pineal Res 69: e12653. 32239546 10.1111/jpi.12653

[bibr13-02698811211032439] Comai S Gobbi G (2014) Unveiling the role of melatonin MT2 receptors in sleep, anxiety and other neuropsychiatric diseases: a novel target in psychopharmacology. J Psychiatry Neurosci 39: 6–21. 23971978 10.1503/jpn.130009 PMC3868666

[bibr14-02698811211032439] Comai S Ochoa-Sanchez R Gobbi G (2013) Sleep-wake characterization of double MT(1)/MT(2) receptor knockout mice and comparison with MT(1) and MT(2) receptor knockout mice. Behav Brain Res 243: 231–238. 23333399 10.1016/j.bbr.2013.01.008

[bibr15-02698811211032439] Conti A Conconi S Hertens E , et al. (2000) Evidence for melatonin synthesis in mouse and human bone marrow cells. J Pineal Res 28: 193–202. 10831154 10.1034/j.1600-079x.2000.280401.x

[bibr16-02698811211032439] Cornblatt BA Lenzenweger MF Erlenmeyer-Kimling L (1989) The continuous performance test, identical pairs version: II. Contrasting attentional profiles in schizophrenic and depressed patients. Psychiatry Res 29: 65–85. 2772099 10.1016/0165-1781(89)90188-1

[bibr17-02698811211032439] Crick F (1984) Function of the thalamic reticular complex: The searchlight hypothesis. Proc Natl Acad Sci U S A 81: 4586–4590. 6589612 10.1073/pnas.81.14.4586 PMC345636

[bibr18-02698811211032439] Davis MT DellaGiogia N Maruff P , et al. (2021) Acute cognitive effects of single-dose intravenous ketamine in major depressive and posttraumatic stress disorder. Transl Psychiatry 11:205. 33833217 10.1038/s41398-021-01327-5 PMC8032778

[bibr19-02698811211032439] Dawson N Morris BJ Pratt JA (2011) Subanaesthetic ketamine treatment alters prefrontal cortex connectivity with thalamus and ascending subcortical systems Schizophr Bull 39: 366–377. 22114100 10.1093/schbul/sbr144 PMC3576175

[bibr20-02698811211032439] Dollins AB Lynch HJ Wurtman RJ , et al. (1993) Effect of pharmacological daytime doses of melatonin on human mood and performance. Psychopharmacology 112: 490–496. 7871062 10.1007/BF02244899

[bibr21-02698811211032439] Dubocovich ML (2007) Melatonin receptors: Role on sleep and circadian rhythm regulation. Sleep Med 8(Suppl. 3): 34–42. 18032103 10.1016/j.sleep.2007.10.007

[bibr22-02698811211032439] Ferguson BR Gao W-J (2018) Thalamic control of cognition and social behavior via regulation of gamma-aminobutyric acidergic signaling and excitation/inhibition balance in the medial prefrontal cortex. Biol Psychiatry 83: 657–669. 29373121 10.1016/j.biopsych.2017.11.033 PMC5862785

[bibr23-02698811211032439] Ferrarelli F Tononi G (2011) The thalamic reticular nucleus and schizophrenia. Schizophr Bull 37: 306–315. 21131368 10.1093/schbul/sbq142 PMC3044616

[bibr24-02698811211032439] Fitzgibbon T Kikuchi N (2011) Projections from cingulate cortex to the cat’s thalamic reticular nucleus. Vis Neurosci 28: 433–444. 21880166 10.1017/S0952523811000319

[bibr25-02698811211032439] Fletcher PJ Soko AD Higgins GA (2013) Impulsive action in the 5-choice serial reaction time test in 5-HT₂c receptor null mutant mice. Psychopharmacology 226: 561–570. 23192316 10.1007/s00213-012-2929-0

[bibr26-02698811211032439] Gomez-Corvera A Cerrillo I Molinero P , et al. (2009) Evidence of immune system melatonin production by two pineal melatonin deficient mice, C57BL/6 and Swiss strains. J Pineal Res 47: 15–22. 19522737 10.1111/j.1600-079X.2009.00683.x

[bibr27-02698811211032439] Goto M Oshima I Tomita T , et al. (1989) Melatonin content of the pineal gland in different mouse strains. J Pineal Res 7: 195–204. 2769571 10.1111/j.1600-079x.1989.tb00667.x

[bibr28-02698811211032439] Guillery RW Feig SL Lozsadi DA (1998) Paying attention to the thalamic reticular nucleus. Trends Neurosci 21: 28–32. 9464683 10.1016/s0166-2236(97)01157-0

[bibr29-02698811211032439] Gunn PJ Middleton B Davies SK , et al. (2016) Sex differences in the circadian profiles of melatonin and cortisol in plasma and urine matrices under constant routine conditions. Chronobiol Int 33: 39–50. 26731571 10.3109/07420528.2015.1112396 PMC4819823

[bibr30-02698811211032439] Heisler LK Zhou L Bajwa P , et al. (2007) Serotonin 5-HT(2C) receptors regulate anxiety-like behavior. Genes Brain Behav 6: 491–496. 17451451 10.1111/j.1601-183X.2007.00316.x

[bibr31-02698811211032439] Ho J Tumkaya T Aryal S , et al. (2019) Moving beyond P values: Data analysis with estimation graphics. Nat Methods 16: 565–566. 31217592 10.1038/s41592-019-0470-3

[bibr32-02698811211032439] Horikoshi M Yaghootkar H Mook-Kanamori DO , et al. (2013) New loci associated with birth weight identify genetic links between intrauterine growth and adult height and metabolism. Nat Genet 45: 76–82. 23202124 10.1038/ng.2477 PMC3605762

[bibr33-02698811211032439] Horne JA Anderson NR Wilkinson RT (1983) Effects of sleep deprivation on signal detection measures of vigilance: Implications for sleep function. Sleep 6: 347–358. 6665397 10.1093/sleep/6.4.347

[bibr34-02698811211032439] Hsieh PC Chu CL Yang YK , et al. (2005) Norms of performance of sustained attention among a community sample: Continuous performance test study. Psychiatry Clin Neurosci 59: 170–176. 15823163 10.1111/j.1440-1819.2005.01353.x

[bibr35-02698811211032439] Hutchinson AJ Hudson RL Dubocovich ML (2012) Genetic deletion of MT(1) and MT(2) melatonin receptors differentially abrogates the development and expression of methamphetamine-induced locomotor sensitization during the day and the night in C3H/HeN mice. J Pineal Res 53: 399–409. 22672659 10.1111/j.1600-079X.2012.01010.x PMC3568497

[bibr36-02698811211032439] Hvoslef-Eide M Nilsson SR Hailwood JM , et al. (2018) Effects of anterior cingulate cortex lesions on a continuous performance task for mice. Brain Neurosci Adv 2: 2398212818772962. 31168482 10.1177/2398212818772962 PMC6546594

[bibr37-02698811211032439] Ito M Kanno M Mori Y , et al. (1997) Attention deficits assessed by continuous performance test and span of apprehension test in Japanese schizophrenic patients. Schizophr Res 23: 205–211. 9075298 10.1016/s0920-9964(96)00108-9

[bibr38-02698811211032439] Kamal M Gbahou F Guillaume JL , et al. (2015) Convergence of melatonin and serotonin (5-HT) signaling at MT2/5-HT2C receptor heteromers. J Biol Chem 290: 11537–11546. 25770211 10.1074/jbc.M114.559542 PMC4416857

[bibr39-02698811211032439] Katz R De Sanctis P Mahoney JR , et al. (2010) Cognitive control in late-life depression: Response inhibition deficits and dysfunction of the anterior cingulate cortex. Am J Geriatr Psychiatry 18: 1017–1025. 20808083 10.1097/JGP.0b013e3181d695f2 PMC3770530

[bibr40-02698811211032439] Kennaway DJ (2019) Melatonin research in mice: A review. Chronobiol Int 36: 1167–1183. 31198062 10.1080/07420528.2019.1624373

[bibr41-02698811211032439] Kim CH Hvoslef-Eide M Nilsson SR , et al. (2015) The continuous performance test (rCPT) for mice: A novel operant touchscreen test of attentional function. Psychopharmacology 232: 3947–3966. 26415954 10.1007/s00213-015-4081-0 PMC4600477

[bibr42-02698811211032439] Kishi T Nomura I Sakuma K , et al. (2019) Melatonin receptor agonists-ramelteon and melatonin-for bipolar disorder: A systematic review and meta-analysis of double-blind, randomized, placebo-controlled trials. Neuropsychiatr Dis Treat 15: 1479–1486. 31239683 10.2147/NDT.S198899 PMC6553999

[bibr43-02698811211032439] Lacoste B Angeloni D Dominguez-Lopez S , et al. (2015) Anatomical and cellular localization of melatonin MT1 and MT2 receptors in the adult rat brain. J Pineal Res 58: 397–417. 25726952 10.1111/jpi.12224

[bibr44-02698811211032439] Larson J Jessen RE Uz T , et al. (2006) Impaired hippocampal long-term potentiation in melatonin MT2 receptor-deficient mice. Neurosci Lett 393: 23–26. 16203090 10.1016/j.neulet.2005.09.040

[bibr45-02698811211032439] Lee MD Wagenmakers EJ (2013) Bayesian Cognitive Modeling: A Practical Course. Cambridge: Cambridge University Press.

[bibr46-02698811211032439] Liu J Clough SJ Dubocovich ML (2017) Role of the MT1 and MT2 melatonin receptors in mediating depressive- and anxiety-like behaviors in C3H/HeN mice. Genes Brain Behav 16: 546–553. 28160436 10.1111/gbb.12369

[bibr47-02698811211032439] Liu J Clough SJ Hutchinson AJ , et al. (2016) MT1 and MT2 Melatonin Receptors: A therapeutic perspective. Annu Rev Pharmacol Toxicol 56: 361–383. 26514204 10.1146/annurev-pharmtox-010814-124742 PMC5091650

[bibr48-02698811211032439] Liu SK Hwu H-G Chen WJ (1997) Clinical symptom dimensions and deficits on the continuous performance test in schizophrenia. Schizophr Res 25: 211–219. 9264176 10.1016/s0920-9964(97)00026-1

[bibr49-02698811211032439] Macdonald KD Fifkova E Jones MS , et al. (1998) Focal stimulation of the thalamic reticular nucleus induces focal gamma waves in cortex. J Neurophysiol 79: 474–477. 9425216 10.1152/jn.1998.79.1.474

[bibr50-02698811211032439] Mass R Wolf K Wagner M , et al. (2000) Differential sustained attention/vigilance changes over time in schizophrenics and controls during a degraded stimulus Continuous Performance Test. Eur Arch Psychiatry Clin Neurosci 250: 24–30. 10738861 10.1007/pl00007535

[bibr51-02698811211032439] McAlonan K Cavanaugh J Wurtz RH (2006) Attentional modulation of thalamic reticular neurons. J Neurosci 26: 4444–4450. 16624964 10.1523/JNEUROSCI.5602-05.2006 PMC6674014

[bibr52-02698811211032439] Millan MJ (2005) Serotonin 5-HT2C receptors as a target for the treatment of depressive and anxious states: Focus on novel therapeutic strategies. Therapies 60: 441–460. 10.2515/therapie:2005065 16433010

[bibr53-02698811211032439] Mitchell EJ Brett RR Armstrong JD , et al. (2020a) Temporal dissociation of phencyclidine: Induced locomotor and social alterations in rats using an automated homecage monitoring system – implications for the 3Rs and preclinical drug discovery. J Psychopharmacol 34: 709–715. 32438848 10.1177/0269881120920455 PMC7675779

[bibr54-02698811211032439] Mitchell EJ Thomson DM Openshaw RL , et al. (2020b) Drug-responsive autism phenotypes in the 16p11.2 deletion mouse model: A central role for gene-environment interactions. Sci Rep 10: 12303. 32704009 10.1038/s41598-020-69130-8 PMC7378168

[bibr55-02698811211032439] Miyata A Iwamoto K Kawano N , et al. (2015) The effects of acute treatment with ramelteon, triazolam, and placebo on driving performance, cognitive function, and equilibrium function in healthy volunteers. Psychopharmacology 232: 2127–2137. 25533998 10.1007/s00213-014-3843-4

[bibr56-02698811211032439] Ng KY Leong MK Liang H , et al. (2017) Melatonin receptors: distribution in mammalian brain and their respective putative functions. Brain Struct Funct 222: 2921–2939. 28478550 10.1007/s00429-017-1439-6

[bibr57-02698811211032439] Ochoa-Sanchez R Comai S Lacoste B , et al. (2011) Promotion of non-rapid eye movement sleep and activation of reticular thalamic neurons by a novel MT2 melatonin receptor ligand. J Neurosci 31: 18439–18452. 22171046 10.1523/JNEUROSCI.2676-11.2011 PMC6623882

[bibr58-02698811211032439] Ochoa-Sanchez R Rainer Q Comai S , et al. (2012) Anxiolytic effects of the melatonin MT(2) receptor partial agonist UCM765: Comparison with melatonin and diazepam. Prog Neuropsychopharmacol Biol Psychiatry 39: 318–325. 22789661 10.1016/j.pnpbp.2012.07.003

[bibr59-02698811211032439] O’Neal-Moffitt G Pilli J Kumar SS , et al. (2014) Genetic deletion of MT(1)/MT(2) melatonin receptors enhances murine cognitive and motor performance. Neuroscience 277: 506–521. 25046530 10.1016/j.neuroscience.2014.07.018

[bibr60-02698811211032439] Osipova D Takashima A Oostenveld R , et al. (2006) Theta and gamma oscillations predict encoding and retrieval of declarative memory. J Neurosci 26: 7523–7531. 16837600 10.1523/JNEUROSCI.1948-06.2006 PMC6674196

[bibr61-02698811211032439] Papp M Litwa E Gruca P , et al. (2006) Anxiolytic-like activity of agomelatine and melatonin in three animal models of anxiety. Behav Pharmacol 17: 9–18. 16377959 10.1097/01.fbp.0000181601.72535.9d

[bibr62-02698811211032439] Parnaudeau S O Neill P-K Bolkan Scott S , et al. (2013) Inhibition of mediodorsal thalamus disrupts thalamofrontal connectivity and cognition. Neuron 77: 1151–1162. 23522049 10.1016/j.neuron.2013.01.038 PMC3629822

[bibr63-02698811211032439] Parnaudeau S Taylor K Bolkan SS , et al. (2015) Mediodorsal thalamus hypofunction impairs flexible goal-directed behavior. Biol Psychiatry 77: 445–453. 24813335 10.1016/j.biopsych.2014.03.020 PMC4177020

[bibr64-02698811211032439] Pinault D Deschênes M (1992) Voltage-dependent 40-Hz oscillations in rat reticular thalamic neurons in vivo. Neuroscience 51: 245–258. 1465191 10.1016/0306-4522(92)90312-p

[bibr65-02698811211032439] Pratt JA Dawson N Morris BJ , et al. (2017) Thalamo-cortical communication, glutamatergic neurotransmission and neural oscillations: A unique window into the origins of ScZ? Schizophr Res 180: 4–12. 27317361 10.1016/j.schres.2016.05.013

[bibr66-02698811211032439] Pratt JA Morris BJ (2015) The thalamic reticular nucleus: A functional hub for thalamocortical network dysfunction in schizophrenia and a target for drug discovery. J Psychopharmacol 29: 127–137. 25586397 10.1177/0269881114565805

[bibr67-02698811211032439] Quintana DS Williams DR (2018) Bayesian alternatives for common null-hypothesis significance tests in psychiatry: A non-technical guide using JASP. BMC Psychiatry 18: 178. 29879931 10.1186/s12888-018-1761-4 PMC5991426

[bibr68-02698811211032439] Regenass W Moller M Harvey BH (2018) Studies into the anxiolytic actions of agomelatine in social isolation reared rats: Role of corticosterone and sex. J Psychopharmacol 32: 134–145. 29082818 10.1177/0269881117735769

[bibr69-02698811211032439] Robinson ES Dalley JW Theobald DE , et al. (2008) Opposing roles for 5-HT2A and 5-HT2C receptors in the nucleus accumbens on inhibitory response control in the 5-choice serial reaction time task. Neuropsychopharmacology 33: 2398–2406. 18046307 10.1038/sj.npp.1301636

[bibr70-02698811211032439] Roseboom PH Namboodiri MA Zimonjic DB , et al. (1998) Natural melatonin ‘knockdown’ in C57BL/6J mice: Rare mechanism truncates serotonin N-acetyltransferase. Brain Res Mol Brain Res 63: 189–197. 9838107 10.1016/s0169-328x(98)00273-3

[bibr71-02698811211032439] Sax KW Strakowski SM McElroy SL , et al. (1995) Attention and formal thought disorder in mixed and pure mania. Biol Psychiatry 37: 420–423. 7772653 10.1016/0006-3223(95)00310-D

[bibr72-02698811211032439] Seidman LJ Van Manen K-J Turner WM , et al. (1998) The effects of increasing resource demand on vigilance performance in adults with schizophrenia or developmental attentional/learning disorders: A preliminary study. Schizophr Res 34: 101–112. 9824882 10.1016/s0920-9964(98)00097-8

[bibr73-02698811211032439] Shirayama Y Takahashi M Suzuki M , et al. (2014) Effects of add-on ramelteon on cognitive impairment in patients with schizophrenia: An open-label pilot trial. Clin Psychopharmacol Neurosci 12: 215–217. 25598825 10.9758/cpn.2014.12.3.215 PMC4293167

[bibr74-02698811211032439] Taylor D Sparshatt A Varma S , et al. (2014) Antidepressant efficacy of agomelatine: Meta-analysis of published and unpublished studies. BMJ 348: g2496. 10.1136/bmj.g1888 PMC3959623 24647162

[bibr75-02698811211032439] ter Horst JP de Kloet ER Schächinger H , et al. (2012) Relevance of stress and female sex hormones for emotion and cognition. Cell Mol Neurobiol 32: 725–735. 22113371 10.1007/s10571-011-9774-2 PMC3377901

[bibr76-02698811211032439] Vivien-Roels B Malan A Rettori MC , et al. (1998) Daily variations in pineal melatonin concentrations in inbred and outbred mice. J Biol Rhythms 13: 403–409. 9783231 10.1177/074873098129000228

[bibr77-02698811211032439] Voikar V Polus A Vasar E , et al. (2005) Long-term individual housing in C57BL/6J and DBA/2 mice: Assessment of behavioral consequences. Genes Brain Behav 4: 240–252. 15924556 10.1111/j.1601-183X.2004.00106.x

[bibr78-02698811211032439] Wade AG Farmer M Harari G , et al. (2014) Add-on prolonged-release melatonin for cognitive function and sleep in mild to moderate Alzheimer’s disease: A 6-month, randomized, placebo-controlled, multicenter trial. Clin Interv Aging 9: 947–961. 24971004 10.2147/CIA.S65625 PMC4069047

[bibr79-02698811211032439] Weil ZM Hotchkiss AK Gatien ML , et al. (2006) Melatonin receptor (MT1) knockout mice display depression-like behaviors and deficits in sensorimotor gating. Brain Res Bull 68: 425–429. 16459197 10.1016/j.brainresbull.2005.09.016

[bibr80-02698811211032439] Wimmer RD Schmitt LI Davidson TJ , et al. (2015) Thalamic control of sensory selection in divided attention. Nature 526: 705–709. 26503050 10.1038/nature15398 PMC4626291

